# Case report: Recurrent lung infections following treatment with pralsetinib for an elderly patient with *RET*-fusion positive NSCLC

**DOI:** 10.3389/fonc.2022.1024365

**Published:** 2022-12-08

**Authors:** Li An, Pengzhi Chen, Junfeng Wang, Xuebing Qin, Tingting Liu, Yanhong Gao, Peng Wang, Dong Zhang, Xiangqun Fang, Zhijian Zhang

**Affiliations:** ^1^ Department of Respiratory and Critical Care Medicine, The Second Medical Center&National Clinical Research Center for Geriatric Disease, Chinese PLA General Hospital, Beijing, China; ^2^ Department of Oncology, The Second Medical Center&National Clinical Research Center for Geriatric Disease, Chinese PLA General Hospital, Beijing, China

**Keywords:** pralsetinib, *RET*-fusion, lung cancer, cryptococcal pneumonia, pulmonary abscess

## Abstract

Patients with *RET* fusions represent 1-2% of all cases of non-small cell lung cancer (NSCLC), the majority of whom are younger, and are extremely rare in the elderly. As a selective *RET* inhibitor, pralsetinib has been shown to be efficacious and well-tolerated in patients with *RET*-fusion NSCLC. Nevertheless, there are currently insufficient data available for assessing the activity and safety of pralsetinib in elderly patients with NSCLC. Herein, we report an 81-year-old NSCLC patient with *KIF5B-RET* fusion, who achieved stable disease for more than 9 months at a low-dose of pralsetinib as second-line therapy. Of particular note, during pralsetinb therapy, his clinical course was complicated by cryptococcal pneumonia and staphylococcus aureus lung abscess. Our study demonstrates that pralsetinib is an effective therapeutic option that provides survival benefits for elderly NSCLC patients harboring *RET* fusion. However, during pralsetinb therapy, treating physicians should maintain particular vigilance for the increased risk of infection, especially in elderly patients.

## Introduction

The activation of Rearranged during transfection (*RET*), a well-known oncogenic driver, can lead to the development of many malignancies (1, 2). So far, *RET* fusions have been reported in approximately 1% to 2% of non-small cell lung cancer (NSCLC), and this molecular subtype of lung cancer is predominantly present in patients of younger age, non-smokers, and non-squamous disease (3–5). Traditionally, treatment for *RET* fusion-positive NSCLC was challenging because conventional therapies such as platinum-based chemotherapy, multikinase inhibitors, and immune checkpoint inhibitors have shown limited efficacy and substantial toxicities (6).

Pralsetinib, a selective *RET* inhibitor, has opened a new era in the precision therapy for *RET* fusion-positive NSCLC. In the global trial ARROW, this drug has manifested striking efficacy and tolerable side effects, with the 61% of response rate in NSCLC patients who had previously received chemotherapy and 70% in previously untreated NSCLC patients (7). Based on the data of ARROW trial, pralsetinib was approved by the United States Food and Drug Association (FDA) on December 1, 2020, and by the National Medical Products Administration (NMPA) of China on March 24, 2021, for the treatment of adults with *RET* fusion-positive NSCLC. Since then, pralsetinib has been increasingly used in clinical practice and has proven to be effective against *RET*-driven NSCLC in a few real-world studies (8–10). Nonetheless, since most of the NSCLC patients enrolled in the ARROW trial and prior clinical studies have been younger than 70 years, there is insufficient information available to determine whether pralsetinib is safe and effective in elderly patients. Therefore, we present a case of NSCLC with *RET* fusion in an 81-year-old male who was treated with pralsetinib as second-line therapy.

## Case presentation

**Figure 1:**
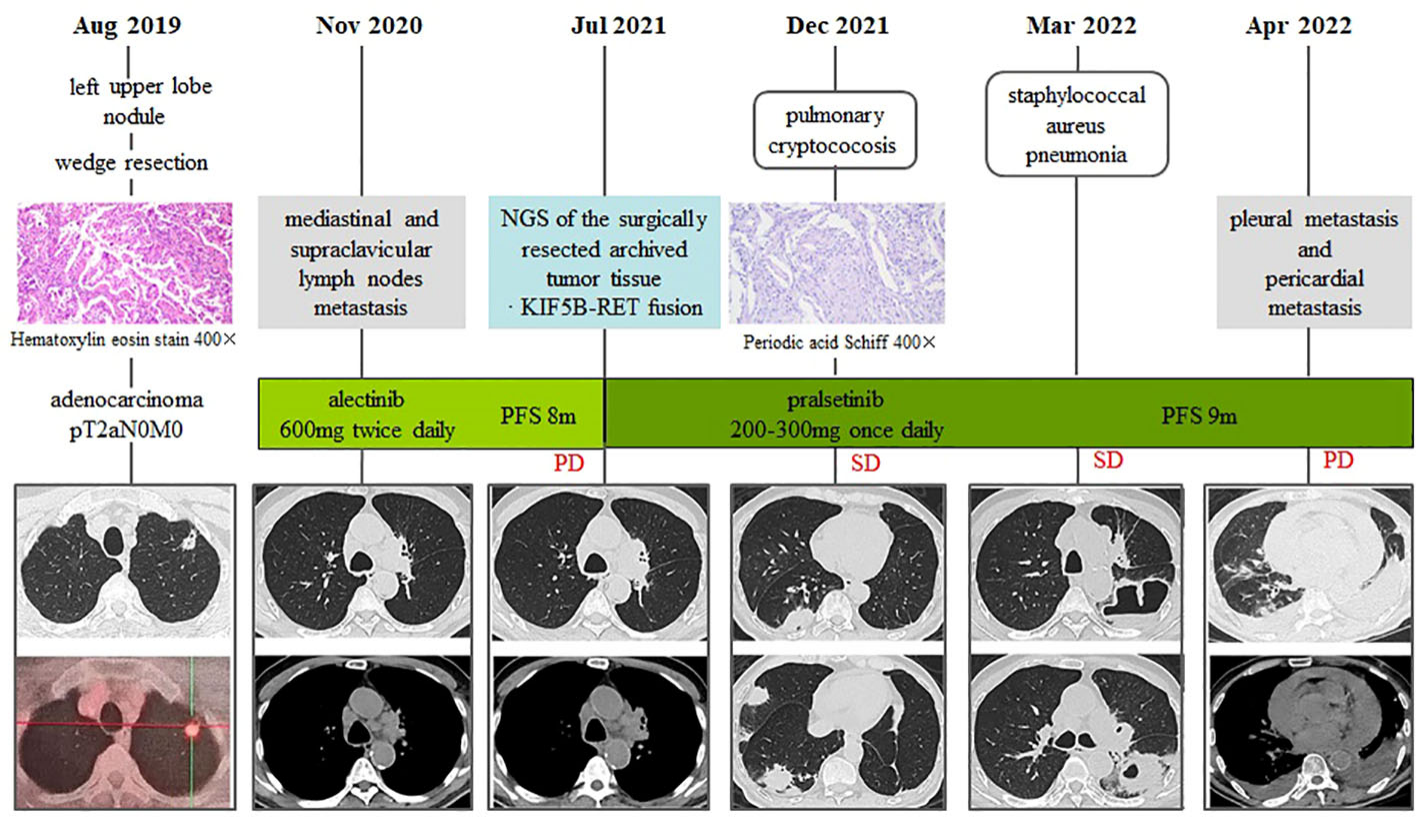
Timeline of clinical course.

An 81-year-old male was admitted to our hospital on August 17, 2019, with an incidental finding of a nodule in the right upper lobe during a physical examination without any clinical symptoms. His past medical history included diabetes mellitus type II, coronary heart disease, and hypertension. He did not smoke cigarettes or drink alcohol. On August 27, 2019, the patient underwent thoracoscopic wedge resection of the left upper lobe and dissection of the mediastinal lymph node. Postoperative pathology confirmed adenocarcinoma (1.8×1.5×1.2cm), with tumor thrombus in the interstitial vessels, and visceral pleura invasion (**Figure 1**). PD-L1 expression was 15%. According to the 8th American Joint Committee on cancer staging system (11), his postoperative staging was pT2aN0M0, stage IB. The patient refused any postoperative adjuvant therapy.

After being discharged from the hospital, the patient received regular follow-up examinations and remained progression-free for 14 months. However, on October 27, 2020, the chest CT scan and the PET-CT revealed metastasis to mediastinal and supraclavicular lymph nodes. The surgically resected archived tumor tissue was sent for next-generation sequencing with a 34-gene lung cancer panel, and *KIF5B-RET* fusion was detected. Additionally, co-alterations were not indentified for *EGFR, ALK, ROS1, MET, HER2, BRAF, and KRAS.* The ECOG performance status score was 2. The patient refused chemotherapy. At that time, there were no targeted therapies approved or available for the treatment of RET-fusion positive NSCLC in the mainland China. In a previous case series study of patients with NSCLC with RET-fusion, alectinib demonstrated clinical activity and good tolerance (12). On the Basis of this information, the patient was treated with alectinib as first-line treatment, which was administered orally at a dose of 600 mg twice daily since November 15, 2020. As measured according to Response Evaluation Criteria in Solid Tumors (RECIST) (13) the patient experienced stable disease after initiation of alectinib treatment for 1 month, 3 months and 6 months, with hypertension as the main adverse effect. In July 2021, disease progression was revealed by the chest CT scan, showing increase in the size of mediastinal and supraclavicular lymph nodes (**Figure 1**). Pralsetinib was initiated with a dose of 400 mg once daily from July 10th, 2021. After two weeks, he experienced grade 2 hypertension, grade 1 suppression of platelets and neutrophils, as well as grade 1 elevation of aspartate aminotransferase (AST). The dosage of pralsetinib was reduced to 300 mg per day but the adverse effects did not relieve significantly. Therefore, since August 15th, 2021, pralsetinib was reduced to 200 mg per day with good tolerance. The CT scan was repeated every month after initiation of pralsetinib and demonstrated a stable disease. Pralsetinib was adjusted to 300 mg once a day in November 2021.

On November 29th, 2021, the follow-up chest CT scan revealed multiple nodules and patchy shadows on the right middle and right lower lobes. The CT-guided percutaneous lung biopsy revealed epithelial granulomas with multinucleated giant cells and cryptococcus spores stained positive with PAS (**Figure 1**). Neurological physical examination and brain magnetic resonance imaging (MRI) revealed no signs of cryptococcal meningitis. Therefore, he was diagnosed as pulmonary cryptococcosis and received fluconazole (450mg po QD) for antifungal therapy since December 14th, 2021. One month later, the chest CT scan showed that the lesions of the original cryptococcal infection in the right middle and lower lung had grown in size. Therefore, voriconazole (200mg po BID) was used as an alternative antifungal treatment.

On March 3, 2022, the patient was hospitalized for one-week history of low-grade fever, cough with purulent sputum and hemoptysis. Laboratory investigations showed white cell count 8.3×10^9^,/l, neutrophils percentage of 80%, and CRP level of 100.5 mg/L. On the chest CT scan, a cavitating mass with an air-fluid level was found in the left lower lobe, but the lesions of cryptococcal infection were partially absorbed (**Figure 1**). The sputum culture revealed methicillin-sensitive staphylococcus aureus (MSSA). The patient received intravenous biapenem to treat MSSA lung abscess. Upon initiation of antibiotic therapy, the patient’s clinical condition improved, and chest CT scan revealed a decrease in cavitating opacity.

On April 7, 2022, the patient complained of dyspnea, palpitations and fatigue, and the chest CT scan revealed pericardial effusion and bilateral pleural effusions (**Figure 1**). We performed pericardiocentesis and thoracocentesis, the drainage of which are both bloody. Cytological examination of the pleural effusion and pericardial effusion revealed adenocarcinoma cells, indicating disease progression with involvements of pleura and pericardium. Distant metastasis was also discovered in the liver through the abdominal CT scan. Since then, the patient’s general condition deteriorated, and he became bedridden, with an ECOG performance status score of 4. To treat his pleural and pericardial effusion, we attempted intrapleural chemotherapy with cisplatin and bevacizumab, as well as pericardial chemotherapy with mitomycin. As a result, his pericardial effusion was effectively controlled, but not his pleural effusion. He developed respiratory distress and his clinical condition rapidly deteriorated. He died on June 21, 2022.

## Discussion

To date, the efficacy and safety of pralsetinib in elderly NSCLC patients have not been adequately investigated. Here, we describe the case of an 81-year-old male with *RET* fusion-positive NSCLC who achieved a PFS of more than 9 months with second-line pralsetinib treatment. However, the patient suffered recurrent lung infection events following the initiation of pralsetinib.

*RET* fusion-driven NSCLC is a rare genomic subtype that occurs mostly in younger patients. In a retrospective study involving 2025 patients with NSCLC, *RET* fusions constitute approximately 1.4% of all cases, with an extremely high incidence among patients below 70 years of age (14). Another retrospective multicenter study revealed that 129 patients with *RET*-rearranged NSCLC had a median age of 57 years (range: 24 to 82) (5). Due to this age distribution characteristics, elderly patients with NSCLC and *RET* fusions are relatively uncommon.

Pralsetinib is a potent and selective *RET* inhibitor that has manifested striking efficacy for *RET*-driven NSCLC patients. In the ARROW clinical trial, the efficacy population included 87 previously platinum-treated patients and 27 treatment-naive patients with advanced NSCLC, the median age was 60 (95%CI: 53-68) years and 65 (95%CI: 54-69) years respectively. Treatment with pralsetinib demonstrated a response rate of 61% and a PFS of 17 months in patients who had previously undergone chemotherapy, and 70% and 9.1 months in treatment-naive patients. Furthermore, a real-world study in Korea included 10 NSCLC patients with a median age of 53 (range: 29-65) years who received pralsetinib therapy. Satisfactory effects have been shown in this study, with partial response in eight patients and disease stabilization in another two patients (8). In addition, pralsetinib was used as neoadjuvant therapy in a case study, in which a 54-year-old female with stage IIIA lung adenocarcinoma achieved apparent radiologic downstaging after pralsetinib therapy for one month, and the mass of primary tumor was reduced by 74% postoperatively (9). Even though pralsetinib has consistently shown therapeutic benefits in previous studies, its effects on elderly NSCLC patients with *RET* fusion remain unclear.

In the current study, pralsetinib treatment in this 81-year-old patient finally led to a PFS of more than 9 months, with the best response of stable disease. The PFS achieved with pralsetinib here was superior to that obtained with traditional therapies, including platinum-based chemotherapy (6.4-7.8 months) (15), multikinase inhibitors (4.5-7.3 months) (16–19), and immune checkpoint inhibition (2.1-3.4 months) (20, 21). Together with the results from previous studies, our data further support pralsetinib as a favorable therapeutic option for patients with NSCLC harboring *RET* fusion, even in elderly patients. In addition, it should be noted that the PFS, in this case, is relatively short compared to those from phase I/II ARROW trials. Due to the different dosage of pralsetinib used in our study and the ARROW trial (200-300 mg/d vs 400 mg/d), there is a possibility that dose-dependent effects play a role. Additionally, it is unclear whether the advanced age and poor performance status could negatively affect the patient’s response to pralsetinib. Subgroup analysis by age and performance status, or an accumulation of cases, will be necessary to clarify this issue.

Pralsetinib was reported to be generally well-tolerated in the ARROW trial, with most of the adverse effects being grade 1-2 in severity. In the current study, the old patient developed grade 2 hypertension, grade 1 neutropenia, and grade 1 elevated AST with pralsetinib therapy at an initial dose of 400 mg once daily, which were alleviated with a dosage reduction to 200-300 mg once daily. Of particular note, the patient developed recurrent lung infections during pralsetinib treatment, including cryptococcal pneumonia and staphylococcus aureus pulmonary abscesses, which complicated his clinical course but were well controlled by antimicrobial therapy. As reported in the ARROW trial, pneumonia occurred in 17% of NSCLC patients who received pralsetinib, with multiple pathogens involved, including various bacteria, pneumocystis jirovecii as well as cytomegalovirus and influenza. Among them, the incidence of serious pneumonia in grade 3/4 was 8%, and more than 4% required dosage reduction or dosage interruption (7). In a recent clinical study, three of 10 NSCLC patients receiving pralsetinib developed infection events, one of which was extrapulmonary tuberculosis, one was herpes zoster and one developed both infections concurrently (8). In view of these data, it is suggested that infectious events may be an significant safety issue for NSCLC patients who are receiving pralsetinib. Moreover, it is important to note that the majority of participants in ARROW trial were younger and had better performance status, there may be a higher incidence of infection in the real world, particularly when pralsetinib is administered to the elderly patients.

In the current study, the patient developed troublesome pulmonary infection events during pralsetinib treatments. Pulmonary cryptococosis is an opportunistic fungal disease caused by cryptococcus, most commonly seen in immunocompromised individuals. Cryptococcus is ubiquitous in the natural enviroment, particularly prevalent in pigeon faeces (22). In this case, the patient had no history of feeding or close contact with pigeons, so breathing dust contaminated with pigeon faeces may have been the probable route of infection. In addition to pulmonary cryptococosis, the patient also suffered pneumonia due to staphylococcus aureus. As one of the most common Gram-positive coccus, staphylococcus aureus is frequently found in the respiratory tract and skin. A persistant nasal colonization of staphylococcus aureus was found in up to 30% of the general population, and diabetes, renal insufficiency, and immunosuppression have been associated with increased staphylococcus aureus colonization (23). Notably, there is evidence that nasal carriage of staphylococcus aureus poses a high risk of developing staphylococcus aureus infections. In our case, a possibility exists that staphylococcus aureus pneumonia of the patient may be caused by endogenous inhalation of staphylococcus aureus colonized in the nasal cavity. Indeed, pralsetinib may not be the only factor contributing to the infectious events in this case. Several host-related factors, including advanced age, diabetes mellitus, along with pralsetinib, are synergistic contributors to the immunocompromised status and the subsequent recurrent lung infections. Consequently, clinicians should maintain a heightened awareness of the increased risk of infections, especially when prescribing pralsetinib to aging patients.

Although pralsetinib is highly selective for *RET* kinase, it has been shown that pralsetinib can also inhibit some non-*RET* kinases, such as JAK1/2, DDR1, FGFR1/2, FLT3, PDGFRb, TRKA, TRKC and VEGF2 (24). Among them, the JAK-mediated intracellular signaling pathways play a crucial role in immunoregulation and in host defense (25, 26). Treatment with JAK inhibitors for autoimmune diseases or cancers has been well documented to be associated with an increased frequency of infection, including herpes zoster, fungal infection, mycobacterial infections as well as reactivation of HBV (27–29). Pralsetinib may therefore predispose patients to infections as a result of their off-target effects on JAK1/2, but the precise mechanism has yet to be determined.

This case report describes real-world experiences of using pralsetinib in elderly patients with RET-fusion positive NSCLC, providing a basis for further studies. Despite of this, some limitations of our study should be mentioned here. Firstly, the immune function of the patient was not regularly monitored, which should be done when managing elderly NSCLC patients especially when receiving anti-cancer treatments. Secondly, only one case was included in this study. Therefore, more studies are required to validate the safety and effectiveness of pralsetinib in NSCLC patients with RET-fusion in elderly patients.

In conclusion, our study provides new insights into the efficacy and safety of pralsetinib in elderly patients with *RET* fusion-positive NSCLC. Pralsetinib treatment, even at a low dose, may have clinical benefits for elderly NSCLC patients. The risk for pneumonia may be increased in NSCLC patients who are receiving pralsetinib, especially for those with advanced age. Therefore, for the elderly NSCLC patients receiving pralsetinb, treating physicians should maintain particular vigilance for the increased risk of infection. In order to resolve this issue, elderly patients treated with pralsetinb should be followed regularly to assess their nutritional status, immune function and biomarkers of infection. Additionally, early identification of the symptoms of infection, accurate detection of the causative pathogen as well as timely treatment of infections are also advised.

## Data availability statement

The datasets presented in this study can be found in online repositories. The names of the repository/repositories and accession number(s) can be found in the article/supplementary material.

## Ethics statement

The studies involving human participants were reviewed and approved by The Second Medical Center, Chinese PLA general hospital. The patients/participants provided their written informed consent to participate in this study.

## Author contributions

All the authors were involved in the clinical management of the patient. LA and PC wrote the manuscript. XF reviewed the manuscript. ZZ designed the study and reviewed the manuscript. All authors contributed to the article and approved the submitted version.
